# Pleiocarpa pycnantha leaves and its triterpenes induce apoptotic cell death in Caco-2 cells *in vitro*

**DOI:** 10.1186/s12906-015-0767-4

**Published:** 2015-07-14

**Authors:** Olubunmi Adenike Omoyeni, Ahmed Hussein, Mervin Meyer, Ivan Green, Emmanuel Iwuoha

**Affiliations:** Department of Chemistry, University of the Western Cape, Bellville, South Africa; Department of Biotechnology, Apoptosis Research Unit, University of the Western Cape, Bellville, South Africa

**Keywords:** Apoptosis, colon cancer, Triterpenes, *Pleiocarpa pycnantha*, Caspases, 27- p-*Z*-coumaroloxy ursolic acid, 27- p-*E*-coumaroloxy ursolic acid

## Abstract

**Background:**

Recently, we reported that the crude fractions and pure triterpenes; ursolic acid (**C1**), 27-*E* and 27-*Z* p-coumaric esters of ursolic acid (**C2**, **C3**), together with a new triterpene 2,3-seco-taraxer-14-en-2,3-lactone [pycanocarpine (**C4**)] and its hydrolysed derivative - (2,3-seco-taraxen-4-hydroxy-14-en-2-oic acid) [pycanocarpene (**C5**)] from *Pleiocarpa pycnantha* leaves inhibit cell proliferation. However, there has not been any specific report on the use of *Pleiocarpa pycnantha* leaves and its constituents to kill colorectal adenocarcinoma cancer CaCo-2 cells.

We performed *in vitro* study to evaluate the cytotoxic properties of the ethanolic extract of *P. pycnantha***P**, compounds **C2** and **C3**. A preliminary study of the potential mechanisms were also undertaken.

**Methods:**

Cell viability was measured by WST-1 assay. The Apoptosis level was evaluated by staining with APOPercentage™ dye and the induction of caspases 3/7 and 9 using Caspase-Glo® assays.

**Results:**

The exposure of an ethanolic extract from the leaves of *P. pycnantha* (0.1–1000 μg/ml) and the isolated compounds **C2** and **C3** (6,25–100 μg/ml) to human colorectal cancer cells reduced the cell viability with an IC_50_ > 100, 40.9, 36.3 μg/ml for **P**, **C2** and **C3** respectively, after 24 h of incubation. The APOPercentage^TM^ assay also showed a considerable increase in the percentage of apoptotic cells after 24 h; (25–38 % for **P**, 5–23 % for **C2** and 6–47 % for **C3**). Caspase 3 was also activated which is a hallmark of apoptosis.

**Conclusion:**

These findings suggest that the *P. pycnantha* and the isolated compounds induce cell apoptosis in human colorectal adenocarcinoma cells. A further study with other cell lines is also recommended.

## Background

Cancer is a major public health burden to both the developed and the developing nation.

The global distribution of cancer and the type of cancer that predominates continues to change, especially in economically developing countries. The low- and middle- income countries accounted for about (51 %) of all cancers worldwide in 1975 with this proportion increasing to 55 % in 2007 and is projected to reach 61 % by 2050 [1]. Cancers of the lung, breast, colon/rectum and prostate are no longer largely restricted to the Western industrialized countries but are among the most common cancers worldwide [2].

Neoplasms are the main cause of death worldwide. Each year tumors are diagnosed in about 11 million people, ending with death in 7.6 million; the number forecasted for 2030 is in the region of 13.1 million [3].

Colorectal cancer is the third most common cancer and the third leading cause of cancer death in men and women in the United States. The American Cancer Society estimates that 136,830 people will be diagnosed with colorectal cancer and 50,310 people would have died from the disease in 2014 [4, 5]. The major treatment regimen for cancer are chemotherapy and radiotherapy, which unfortunately have often proved to be toxic to other living cells in the body. Therefore, numerous studies have focused on the application of natural products to prevent and to treat cancer [6].

Natural products have also served as important chemical prototypes for the discovery of new molecules, and continue to be the most promising source of drug leads, especially in the anticancer field [7]. Among bioactive compounds, there is an important group known as the triterpenes and these have demonstrated cytotoxic properties against tumor cells on the one hand while on the other hand display low cytotoxicity towards normal cells [8].

More than 20,000 triterpenes has been isolated and identified from nature, which belong to different chemical groups such as squalene, lanostane, dammarane, lupane, oleanane, ursane, hopane, or triterpenoid sapogenins as for example, cycloartane, friedelane, filicane and cucurbitane triterpenoids [9]. Several biological activities have been reported for triterpenes, which includes; anti-cancer, anti-obesity, anti-diabetic, anti-inflammatory, anti-oxidative, anti-viral, anti-bacterial, and anti-fungal properties [10–20].

Apoptosis is a controlled process of programmed cell death and plays an important role in many normal functions ranging from fetal development to adult tissue homeostasis [21]. Tumors are characterized by uncontrolled proliferation and reduced apoptosis. The activation of apoptotic pathways is a key mechanism by which cytotoxic drugs kill cancer cells. Compounds that block or suppress the growth of tumor cells by inducing apoptosis are considered to have potential as anti-tumoral agents [22].

We have reported on the cytotoxicity of *P. pycnantha* leaves and its triterpenoid contents on HeLa, HT-29, MCF-7 and KMST-6 [23]. We also discovered that the literature is very scarce on the anticancer activity of 27-*E* p-coumaroloxy ursolic acid (**C2**) and 27- *Z p*-coumaroloxy ursolic acid (**C3**), and hence the need for this research. To the best of our knowledge, this is the first report of *P. pycnantha* leaves and its constituents on the cytotoxicity, apoptosis and the molecular mechanisms on colorectal adenocarcinoma (Caco-2 cells).

## Methods

### Plant collection and identification

*Pleiocapa pycnantha* (K. Schum.) Stapf leaves were collected at Ikere Ekiti, Ekiti State, South-West, Nigeria in December, 2010. The botanical identification was done by Femi Omotayo of the Herbarium section of Plant Science Department of Ekiti State University, Ado-Ekiti, Nigeria, where a voucher specimen e-Herbarium UHAA 45 was deposited.

### Extraction and isolation

The ethanolic extract of *Pleiocarpa pycnantha* leaves (P) and compounds C2 (27-*E* p-coumaroloxyursolic acid) and C3 (27-*Z* p-coumaroloxyursolic acid) were obtained as previously described by Omoyeni et al., [23]. Briefly, the ground air-dried leaves of *Pleiocarpa pycnantha* (~1.0 kg) was extracted by cold maceration using 95 % ethanol for 3 days to obtain 81.0 g. About (62.0 g) of the ethanol extract was adsorbed in silica gel and ran on silica gel open column using hexane/EtOAc of varying polarities to obtain 13 fractions labelled as P1-P13. Fractions P4, P7, P8, P9 and P12 displayed cytotoxic activities, varying from strong to moderate activities.

**Fig. 1:**
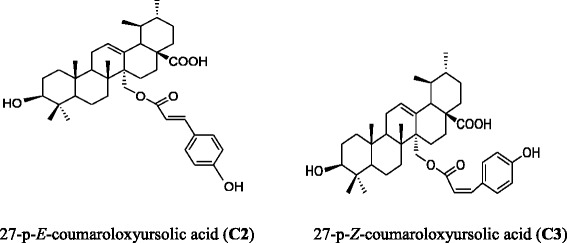
Chemical structure of compounds C2 and C3 [12]

Fraction P12 (5.2 g) was further chromatographed on silica gel column using EtOAc/hexane (50:50–100:0) to afford sub-fraction A-H. The sub-fraction P12E (140 mg) was further purified on sephadex LH-20 column using DCM/MeOH(95:5) and HPLC (MeOH/H2O, 80:20) to afford compound **C2** (5.5 mg) and **C3** (7.3 mg); their chemical structures are illustrated in Fig. 1.

### Chemicals and reagents

Ethanol, dimethyl sulfoxide (DMSO) penicillin-streptomycin and potassium iodide (PI) were purchased from Sigma-Aldrich (St. Louis, MO, USA). DMEM was purchased from Gibco, USA, Fetal bovine serum from Roche, US and trypsin from Invitrogen, Grand Island, New York. Tissue culture flasks, 12 and 96-well plates were obtained from TPP (Trasadingan, Switzerland). APOPercentage™ dye was obtained from Biocolor, UK. Caspase 9 and 3/7 were purchased from Promega, Madison, WI, USA. The WST-1 tetrazolium dye was obtained from (Roche Diagnostics GmbH, Mannheim, Germany).

### Cell culture

The Caco-2 (human colorectal adenocarcinoma) cell line was obtained from American Type Culture Collection (ATCC; Manassas, VA, USA). The cells were maintained in a 37 °C humidified incubator with 5 % CO_2_ saturation. The cells were further maintained in Dulbecco’s Modified Eagle’s medium containing 10 % fetal bovine serum, and 1 % penicillin-streptomycin. All cell culture reagents were obtained from Invitrogen Ltd. (Grand Island, New York). Cells were either plated in 6-well cell culture plates at a cell density of 2.5 × 10^5^ cells per well or in 24 well cell culture plates at a cell density of 1 × 10^5^ cells per well or in a 96-well cell culture plates at a cell density of 2 × 10^4^ cells per well.

### Cell viability assay

Cells were seeded in 96-well culture plates at a density of 2 × 10^4^ cells/well and incubated at 37 °C for 24 h. The next day, cells were exposed to several concentrations of P (0.1–1000 μg/ml), C2 (6,25–100 μg/ml), and C3 (6,25–100 μg/ml). These were further incubated for 24 h, after which the cell viability was measured using the WST-1 assay. The WST-1 reagent (10 μl) was added to each well and incubated for 4 h at 37 °C under 5 % CO_2_ in a humidified incubator. The plates were shaken for 1 min on a shaker and the absorbance of the samples measured at 450 nm (reference wavelength was 750 nm) using a Promega Micro-plate (Madison, WI, USA). Cytotoxicity was expressed as a percentage of the absorbance measured in control untreated cells. IC_50_ values were calculated using Prism Graph pad software. Triplicates experiment and the results expressed as mean ± SEM [23].

### APOPercentage™ assay

The induction of apoptosis was assessed using the APOPercentage assay (Biocolor Ltd., UK). The cells were plated in 24 well cell culture plates at a density of 1 × 10^5^ cells per well. After 24 h the spent medium was replaced with fresh medium containing 10–100 μg/ml of the extract P and 12.5–50 μg/ml of the isolated compounds (C2 and C3). The extract and compounds were dissolved in DMSO prior to adding it to culture medium. The final concentration of DMSO in the treated wells was lower than 1 % (v/v). As a negative control, cells were left untreated, while cells treated with 50 μM cisplatin (a known inducer of apoptosis) served as a positive control. All treatments were done in triplicate. The cells were treated for 24 h, after which the cells were harvested by gentle trypsinization. The cells were stained with the APOPercentage™ dye and analysed by flow cytometry on a Becton Dickinson FACScan instrument (BD Pharmingen™, USA) as described by Meyer et al., [24].

### Caspase 3/7 and 9 assay

Caspases 9 and 3/7 activity was measured using the Caspase-Glo® 9 and Caspase-Glo® 3/7 assays (Promega Corp., USA) according to the method described by Chakravarti et al., [25] with slight modifications. Caco-2 cells were plated in 96-well cell culture plates and treated for 6, 12 and 24 h with 10–100 μg/ml of extract P and 12.5–25 μg/ml of C2, C3 and evaluated for caspases 9 and 3/7 activities. After treatments, the cells were lysed and the cleavage of the substrate by caspases was measured by the generated luminescent signal with a 96 multi-well Glomax luminometer (Promega Corporation, USA).

### Statistical analysis

Data were represented as mean ± SEM of at least three independent experiments. Data were analyzed using Prism Graph Pad software (San Diego, USA). Statistical test two-way ANOVA and Bonferroni post hoc test were conducted for pairwise comparisons. *P* value less than 0.05 was considered statistically significant.

## Results

### Extract P, compounds C2 and C3 decreased cell viability

**Fig. 2:**
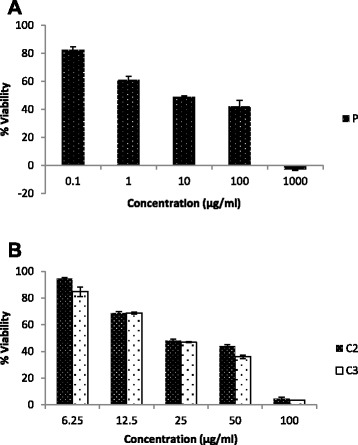
Effect of *Pleiocarpa pycnantha* and isolated compounds on the viability of Caco-2 cells. **a** Cells were treated with various concentrations of ethanol extract of *P. pycnantha* P and the relative cell viability was assessed by WST-1 assay for 24 h. **b** Cells were treated with various concentrations of compound C2 and C3, the relative cell viability was assessed by WST-1 assay for 24 h. The results represent the mean ± SEM of three independent experiments

*P. pycnantha* extract (P), isolated compounds (C2 and C3) decreased cell viability of Caco-2 cells. We examined the effect of *P. pycnantha* ethanolic extract and compounds C2 and C3 in cell viability using WST-1 assay. Caco-2 cells were treated with various concentrations of the extract and compounds C2 and C3, their viability was determined by the uptake of the formazan dye and expressed as percent of untreated control cells. The extract and the compounds induced a dose-dependent increase in viable formazan accumulating cells after the treatment (Fig. 2a and b). The 50 % growth inhibition concentration IC_50_ obtained after 24 h of incubation were > 100, 40.9, 36.3 μg/ml for P, C2 and C3 respectively.

### *P. pycnantha* extract (P), isolated compounds (C2 and C3) induced apoptosis of Caco-2 cells

**Fig. 3:**
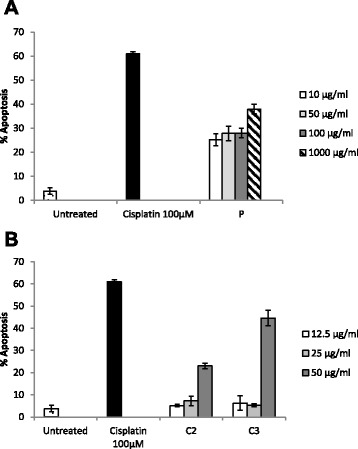
Effect of *Pleiocarpa pycnantha* and isolated compounds on apoptosis in Caco-2 cells. **a** Flow cytometry analysis of APOPercentage™ dye staining after exposure of ethanol extract of *P. pycnantha* (P) treated with various concentrations for 24 h. **b** Cells were treated with increasing concentrations of compound C2 and C3, apoptosis was assessed by APOPercentage™ assay for 24 h as determined by flow cytometry. The results represent the mean ± SEM of three independent experiments

The effect of *P. pycnantha* extract P and compounds C2 and C3 on Caco-2 cell growth was assessed using the APOPercentage ™ dye which detect apoptosis at the stage of phosphatidylserine externalization and its specific for the quantitation of apoptosis. Caco-2 cells were treated for 24 h with different concentrations of P, C2 and C3 and stained with APOPercentage ™ dye and analysed by flow cytometry. The extract P and the tested compounds induced significant levels of apoptosis between (25–38 % for P, 5–23 % for C2 and 6–47 %) in Caco-2 cells (Fig.3a and b). The extract and compounds induce apoptosis in a dose-dependent manner. The cytotoxicity caused by the extract P and compounds C2 and C3 may be due to in part to anti-proliferative and pro-apoptotic effects.

### *P. pycnantha* extract (P), isolated compounds (C2 and C3) induce caspase 3/7 and 9 activity in Caco-2 cells

**Fig. 4:**
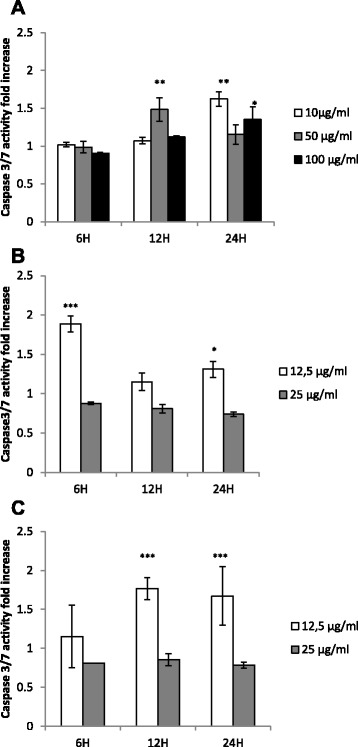
*Pleiocarpa pycnantha* and isolated compounds apoptosis is mediated by caspase3/7 activation. **a** Treatment of cells with various concentration of extract P for 6–24 h (P) treated with various concentrations for 24 h. **b** Cells were treated with increasing concentrations of compound C2 for 6–24 h. **c** Measurement of caspase 3/7 activity when cells were treated with different concentrations of compound C3 for 6–24 h. Data are presented as mean ± SEM,***P < 0.001, **P < 0.01 and *P < 0.05 compared with control

**Fig. 5:**
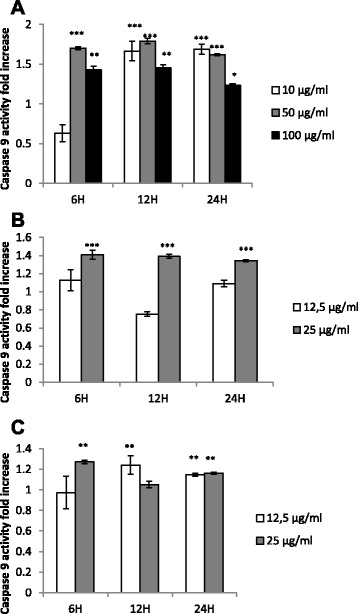
Measurement of caspase 9 activity by *Pleiocarpa pycnantha* and isolated compounds. **a** Treatment of cells with various concentration of extract P for 6–24 h. **b** Cells were treated with increasing concentrations of compound C2 for 6–24 h. **c** Measurement of caspase 9 activity when cells were treated with different concentrations of compound C3 for 6–24 h. Data are presented as mean ± SEM, ***P < 0.001, **P < 0.01 and *P < 0.05 compared with control

The activation of caspases, which are mediators of apoptosis was analysed upon exposure of Caco-2 cells to the *P. pycnantha* ethanol extract and the compounds C2 and C3. The expression of caspase 3/7 and 9 activity was measured in cells exposed to several concentrations of the extract (10–100 μg/ml) for 6–24 h or incubated with C2 and C3 (12.5–25 μg/ml) for 6–24 h. The levels of caspase activation in Caco-2 cells were compared with untreated control cells arbitrarily set to 1.0, the results showed that the extract P significantly increase caspase 3/7 activation at different concentrations and time. At P10 μg/ml, the caspase 3/7 activity reached a maximum (1.62 fold increase at 24 h (Fig.4a). A similar occurrence was observed at P100 μg/ml (1.35 fold increase), while at P50 μg/ml, the maximum caspase activity was recorded at 12 h with approximate fold increase being 1.49 (Fig.4a). Furthermore, compound C2 at 12.5 μg/ml showed a significant caspase 3/7 ≈ 2.0 fold increase activity as early as 6 h while C3 at the same dose had about 1.1 fold increase (Fig.4b and c). At 12 h, compound C3 (12.5 μg/ml) also showed a marked increase caspase activity (1.76 fold increase) as compared with the control, C2 at a similar dose showed only 1.2 fold increase when compared with the control. A similar trend was also observed at 24 h of treatment. Compounds C2 and C3 did not show any significant effect on caspase activation at a higher dose of 25 μg/ml at 6–24 h of treatment (Fig.4b and c). The result of caspase 9 activity of P, C2 and C3 on Caco-2 cells are shown in Fig.5a-c. The result further showed that at concentrations 10–100 μg/ml, P significantly activated caspase 9 at different time points as compared with the untreated control except at 6 h for P10 μg/ml (Fig. 5a). At concentrations, 10, 50, and 100 μg/ml, the maximum caspase 9 activity fold increase obtained were 1.69, 1.79 and 1.45 at 24 h and 12 h respectively. At 25 μg/ml, C2 showed a significant increased caspase 9 activity within 6–24 h of treatment with 1.34–1.41 fold increase (Fig. 5b). Similarly, compound C3 at 25 μg/ml showed an increased caspase 9 activity when compared with the untreated control within 6–24 h from 1.05–1.27 (Fig. 5c). Only a slight increase was observed at the concentration12.5 μg/ml for both C2 and C3 at 6–24 h.

## Discussion

During the past decades, much of effort has been undertaken towards the search for compounds or herbs that kill tumors through induction of apoptosis [26, 27]. Triterpenoids have been reported to exert various pharmacological effects to control a plethora of diseases including cancer [28, 29]. Our previous paper described the cytotoxic properties of these compounds and a crude extract on a panel of cell lines [23]. However their antitumor capacity against colorectal cancer has not been studied.

The present study describes an evaluation of the potential anticancer effects of the crude ethanolic extract P and two pure isolated compounds C2 and C3 from *P. pycnantha.*

Our data illustrates that the extract P and the compounds C2 and C3 demonstrated anti- proliferative and pro-apoptotic effects on Caco-2 colorectal adenocarcinoma cell line in a dose –dependent manner. Using WST-1 assay, we have demonstrated that both extract and the compounds supressed Caco-2 cell viability with C3 displaying a more pronounced activity. The three agents also induce morphological changes, such as cell shrinkage and detachment which were observed under microscope (data not shown).

The anti-proliferative capacity of uvaol, erythrodiol, oleanolic acid and maslinic acid have been reported [30]. It has also been reported that some anticancer agents cause growth inhibition through interfering with the process of the cell cycle [31] while others cause cell death by apoptosis [32]. We further tested the extract P and compounds C2 and C3 for apoptosis using the APOPercentage ^TM^ assay quantified by flow cytometry. The result showed that the percentage of apoptotic cells increased for P, C2 and C3 from 25–38, 5–23 and 6–47 % respectively after 24 h treatments. The execution of apoptosis seems to be uniformly mediated through consecutive activation of the members of a caspase family [33].

We further examined whether the apoptotic effect could be linked to induction of caspase activation. Our data demonstrated that as early as 12 h, P at a concentration of 50 μg/ml showed a significant rise in caspase 3/7 activity while P at a concentration of 10 μg/ml, and P at a high concentration of 100 μg/ml only increased caspase activation after 24 h of treatment. Further to this, compounds C2 at 12.5 μg/ml peaked after 6 h with about 1.89 fold increase while the maximum caspase activity was recorded for C3 at 12.5 μg/ml after 12 h. Since activation of caspase 3 is a hall mark of apoptosis, this is an indication that apoptosis has taken place in the cells. The result obtained from the induction of caspase 9 activity showed that all the concentration of the crude extract P activated caspase 9 except at 6 h for P at 10 μg/ml. There was also a slight activation of caspase 9 at a higher dose of 25 μg/ml for C2 and C3.

## Conclusion

The crude extract P and compounds C2 and C3 showed a significant anti-proliferative effect on Caco-2 cells. To the best of our knowledge, this is the first report on the induction of apoptosis in colorectal adenocarcinoma cells for the extract of *P. pycnantha* and two of its pure compounds. The apoptotic pathway is also characterized by the activation of caspases cascade (vis caspase 9) which culminated in caspase-3 activation. Triterpenoids such as ursolic acid, corosolic acid and maslinic acid have been reported to induce apoptosis by a similar mechanism [28, 29, 34]. However, further studies need to be done regarding alternative mechanisms viz., caspase 8, ROS and cell cycle in order to obtain further clarity about the actual mechanism. Nevertheless, we hope our findings are able to contribute to the development of the crude extract and isolated compounds into viable anticancer agents.

## Abbreviations

WST-1: (4-[3-(4-Iodophenyl)-2-(4-nitrophenyl)-2H-5-tetrazolio]-1,3-benzene disulfonate)
ANOVA: Analysis of variance
DMSO: Dimethyl sulfoxide
PBS: Phosphate buffered saline
